# Analysis of *SOD1* mutations in a Chinese population with amyotrophic lateral sclerosis: a case-control study and literature review

**DOI:** 10.1038/srep44606

**Published:** 2017-03-14

**Authors:** QianQian Wei, QingQing Zhou, YongPing Chen, RuWei Ou, Bei Cao, YaQian Xu, Jing Yang, Hui-Fang Shang

**Affiliations:** 1 Department of Neurology, West China Hospital, Sichuan University, Chengdu, Sichuan, China

## Abstract

Although the copper/zinc superoxide dismutase-1 (*SOD1*) gene has been identified in both familial ALS (FALS) and sporadic ALS (SALS), it has rarely been studied in Chinese patients with ALS, and there are few studies with large samples. This study sought to assess the prevalence of *SOD1* mutations in Chinese ALS patients. We screened a cohort of 499 ALS patients (487 SALS and 12 FALS) from the Department of Neurology at the West China Hospital of Sichuan University and analyzed all coding exons of *SOD1* by Sanger sequencing. In addition, we reviewed the mutation frequencies of common ALS causative genes in Chinese populations. Eight missense mutations in *SOD1* were found in 8 ALS individuals: two novel mutations (p.G73D and p.V120F) and six previously reported mutations. The frequencies of *SOD1* mutations were 1.03% (5/487) in SALS and 25% (3/12) in FALS from Southwest China. A literature review indicated that the mutation rates of major ALS causative genes were 53.55% in FALS and 6.29% in SALS. In Chinese SALS and FALS, the highest mutation frequency was in the *SOD1* gene. Our results suggest that *SOD1* mutation is the most common cause of ALS in Chinese populations and that the mutation spectrum of ALS varies among different ethnic populations.

Amyotrophic lateral sclerosis (ALS) is a genetically heterogeneous neurodegenerative disorder that is clinically characterized by progressive neurological deterioration, the coexistence of upper and lower motor neuron signs, and death from respiratory failure, typically 3–5 years after the onset of symptoms^1^. Most cases of ALS are sporadic, but approximately 10% of cases are familial (FALS)^1^. To date, mutations in 29 genes have been linked to the pathogenesis of ALS^2^. In Caucasians, these mutations are found in a large fraction (approximately 2/3) of FALS patients; however, they are found in only approximately 11% of sporadic ALS (SALS) patients^3^.

The *copper/zinc superoxide dismutase*-*1 (SOD1*) gene is composed of 5 exons coding for a homodimeric enzyme in which each subunit is composed of 154 evolutionarily conserved amino acids and binds with a catalytic Cu^+^ ion and a stabilizing Zn^2+^ ion. The *SOD1* gene was first found to be a causative gene for FALS in 1993^4^. Mutations in the *SOD1* gene account for 12–23.5% of FALS and ~7.3% of apparent SALS, on the basis of multiple large population studies in Caucasians^5^. *SOD1* was reported to be the most commonly mutated ALS gene until a large hexanucleotide (GGGGCC) repeat expansion (HRE) in the chromosome 9 open reading frame 72 (*C9orf72*) gene was identified in 2011. This HRE is found in ~46.4% of FALS cases and ~21.1% of SALS cases, and it is the most common cause of ALS among Caucasian populations^6^. In the past twenty years, other ALS genes have been discovered, including TAR DNA-binding protein (*TARDBP*), Fused in Sarcoma (*FUS*), Angiogenin (*ANG*), Valosin-containing protein (*VCP*), Sequestosome 1 (*SQSTM1*), and Profilin 1 (*PFN1*). However, these genes rarely account for FALS or SALS^7^,^8^. The relative contributions of these ALS causative genes to ALS vary among different populations. Patients with ALS who are from Asia, especially from China, are important components of the rare disease database. However, large-scale screening for mutations of the common causative genes is less common in Chinese patients than in Caucasians. Previous studies have shown that in Chinese SALS patients, the frequency of *TARDBP* is 0–0.93%^9^,^10^,^11^,^12^,^13^,^14^, that of *FUS* is 1.55–1.85%^9^,^11^, that of *ANG* is 0.31%^9^, that of *VCP* is 0%^9^, that of *SQSTM1* is 0.98–1.38%^15^,^16^, that of *PFN1* is 0–0.19%^9^,^17^, that of matrin 3 (*MATR3*) is 0.20%^18^, that of TANK-binding kinase 1 (*TBK1*) is 0.57%^19^ and that of coiled-coil-helix-coiled-coil-helix domain-containing protein 10 (*CHCHD10*) is 0–0.41%^20^,^21^. For the *C9orf72* HRE, five independent studies have revealed that its frequency in SALS is 0–1.53%^9^,^11^,^22^,^23^,^24^. Therefore, these nine common causative genes may account for approximately 6% of Chinese SALS cases. However, regarding *SOD1*, the first identified causative gene, only two studies from northern and central-southern China with a total of 482 SALS patients have investigated the mutation frequency (6/482) of *SOD1* in mainland China^9^,^25^. Thus, the assumption that there are large differences in the frequencies of these causative genes in SALS between Chinese and Caucasian patients may be premature. In the current study, we sought to evaluate the prevalence of *SOD1* mutations in southwest Chinese ALS patients by screening for mutations of the *SOD1* gene in 499 ALS patients and reviewing the mutation frequencies of common ALS causative genes in Chinese populations.

## Methods

### Subjects

This study included a total of 499 Chinese patients diagnosed with ALS (487 with SALS and 12 with FALS) according to the EI Escorial revised criteria^26^ from the Department of Neurology at the West China Hospital of Sichuan University. Detailed patient clinical data, including sex, age of onset, initial symptoms and survival time, were analyzed. Patients with an identifiable family history of ALS among first-, second- or third-degree relatives were classified as having familial ALS (FALS)^27^. A total of 466 unrelated Chinese healthy control subjects (HCs) matched in sex, age, and residential area were included as the control group (46.57% women; mean age 52.78 ± 12.44 years). A neurologist examined all HCs to rule out neurological disorders. This study was approved by the Ethics Committee of Sichuan University and was conducted in accordance with the relevant guidelines. Written informed consent was obtained from all participants.

### Mutation screening

Blood samples were collected from patients and HCs. Genomic DNA was extracted using standard phenol-chloroform protocols. Published primer sequences were used for the amplification of all 5 coding exons and intronic flanking regions of the *SOD1* gene (NM_000454.4)^28^,^29^. The polymerase chain reaction (PCR) products were directly sequenced using an ABI3100 automated DNA sequencing system (Tsingke, Chengdu, China). To determine whether each novel variant was a causative mutation or a neutral polymorphism, the PCR restriction fragment length polymorphism (PCR-RFLP) method was used. The primer sequences, restriction enzymes, and length of PCR products are summarized in Supplementary Table 1. The RFLP results were confirmed by direct sequencing of the PCR products. The detrimental role of the novel mutation was predicted with the Sorting Intolerant from Tolerant (SIFT)^30^ and PolyPhen-2 bioinformatics prediction tools^31^. All the patients who were identified as carrying mutations of the *SOD1* gene were screened for mutations of other common ALS causative genes, including *C9orf72, TARDBP, FUS, PFN1* and *SQSTM1* (Supplementary Tables 2 and 3).

### Literature search

A comprehensive literature review was performed using PubMed (http://www.ncbi.nlm.nih.gov/pubmed/), Medline (National Library of Medicine), and China National Knowledge Internet (www.cnki.net) with the individual search terms “*SOD1*”, “superoxide dismutase 1 gene”, “*FUS*”, “fused in sarcoma”, “*TARDBP*”, “TAR DNA binding protein”, “*ANG*”, “angiogenin”, “*VCP*”, “valosin-containing protein”, “*PFN1*”, “profilin 1”, “*C9orf72*”, “*CHCHD10*”, “*TBK1*”, and “TANK-binding kinase 1”, each combined with “amyotrophic lateral sclerosis” or “ALS” or “motor neuron disease” or “MND” and “Chinese”. To eliminate repeat data, when 2 or more simultaneously published studies had the same first author’s name and similar participant characteristics and data, we included the study with the most comprehensive description of the data. Single case reports were excluded; only case series and case-control studies were included in the review.

### Statistical analysis

Dichotomous variables such as sex and site of onset were analyzed using a standard chi-square test or Fisher’s exact test. Continuous data were compared using Student’s t-test. The results of all continuous data are presented as the mean ± standard deviation (SD), and a two-tailed *p* < 0.05 was considered to be statistically significant. All analyses were performed using SPSS 19.0 (SPSS, Inc., Chicago, IL).

## Results

The demographic and clinical characteristics of patients included in the study are presented in Table 1. In the 499 patients examined, we identified 8 different *SOD1* heterozygous point mutations in 8 ALS patients (Table 2), including 3 FALS and 5 SALS patients. For historical reasons, the numbers of *SOD1* mutations are coded without the initial methionine^32^. For consistency with modern approaches without disregarding the traditional naming convention, we designate the technically correct numbering preceded by “p.”, followed by the traditional naming in parentheses. Thus, the mutations were identified as follows: c.115C > G[p.L39V], c.125G > A[p.G42D], c.131A > G[p.H44R], c.199C > T[p.P67S], c.218G > A[p.G73D], c.335G > A[p.C112Y], c.341T > C[p.I114T] and c.358G > T[p.V120F].

The age of onset in ALS patients carrying *SOD1* mutations ranged from 33.1 years to 59.4 years, and the mean was 45.5 ± 8.5 years. The mean disease duration of five patients with *SOD1* mutations was 25.20 ± 21.81 months (range of 9 to 63 months), excluding the three patients with *SOD1* mutations who were still alive. The eight patients carrying the mutations of the *SOD1* gene in the current study had no mutations in the *C9orf72, TARDBP, FUS, PFN1* and *SQSTM1* genes. The clinical features of patients carrying mutations of the *SOD1* gene are summarized in Table 2.

**Figure 1:**
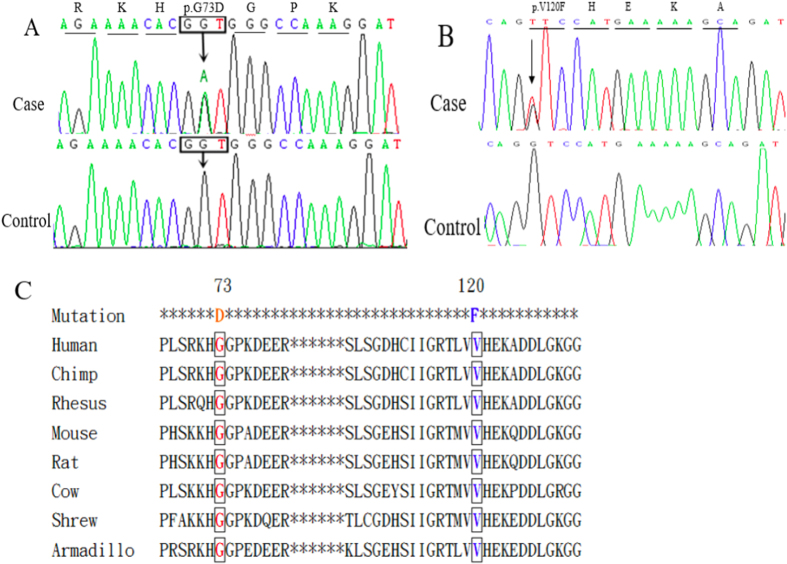
Results of genetic analyses of patients. (**A**) Forward sequence chromatograms of the portions of these PCR products show the heterozygous G > A (p.G73D) in the patient (case 1) but not in the HC. (**B**) Forward sequence chromatograms of the portions of these PCR products show the heterozygous G > T (p.V120F) in the patient (case 2) but not in the HC. (**C**) Protein sequence alignment of SOD1 in vertebrate species and the evolutionary conservation of the SOD1 mutations p.G73D and p.V120F.

The pathogenic roles of p.L39V (L38V), p.G42D (G41D), p.H44R (H43R) and p.I114T (I113T) have been confirmed in ALS with reference to the Single Nucleotide Polymorphism database (dbSNP, http://www.ncbi.nlm.nih.gov/SNP/). In addition, previous studies have reported that p.P67S (P66S) and p.C112Y (C111Y) are likely to be pathogenic^33^,^34^. However, p.G73D and p.V120F had not been reported previously (Fig. 1).

To investigate the contributions of the causative genes for ALS in Chinese patients, we reviewed the previously reported mutation frequencies of common causative genes, including *SOD1, TARDBP, FUS, ANG, VCP, SQSTM1, PFN1, TBK1, CHCHD10* and *C9orf72*, in Chinese populations. The mutation rates of these ALS causative genes were 53.55% in FALS and 6.29% in SALS. In the SALS patients, we found that the highest mutation frequency was in the *SOD1* gene, followed by the *FUS, SQSTM1, TBK1, C9orf72, TARDBP, ANG, CHCHD10, PFN1* and *VCP* genes. In the FALS patients, the highest mutation frequency was also found in the *SOD1* gene, followed by the *TARDBP, FUS* and *C9orf72* genes (Supplementary Table 4).

## Discussion

Although studies on *SOD1* gene mutations in ALS are numerous, to our knowledge, this is the first study on the mutation frequency and clinical features of patients with *SOD1* gene mutations in a large Chinese cohort. In this study, the frequency of *SOD1* gene mutations was found to be 25% (3/12) in FALS and 1.03% (5/487) in SALS patients from southwest China.

Together with the findings of the current study, we found that the *SOD1* gene was the most common causative gene, accounting for 1.45% of SALS and 25.33% of FALS in Chinese patients. However, the proportion was lower than that in Caucasians, especially in SALS (0–7.3%, average 3.06% [35/1142])^5^. In addition, *C9orf72* was the most common gene in Caucasians (SALS: 0–21.1%, average 7.11%[230/3237]; FALS: 21.7–57.9%, average 39.31%[217/562])^6^, but it contributed to only 0.53% of SALS and 5.98% of FALS in Chinese patients, a frequency even lower than that of *TARDBP* or *FUS* (Supplementary Table 4). Therefore, the two most common causative genes, *SOD1* and *C9orf72*, accounted for approximately 2% of SALS and 31% of FALS in Chinese patients and for approximately 10% of SALS and 60% of FALS in Caucasians. This difference in the genetic backgrounds prompted us to search for potential causative genes that account for a large proportion of ALS cases in Chinese patients. Based on these findings, we screened the sequences of the most common causative genes other than *C9orf72* and *SOD1* – *FUS* and *SQSTM1* – in Chinese ALS patients. In addition, due to the small sample of FALS subjects recruited in all included Chinese studies, the mutation frequency may have some bias. Therefore, a large sample of familial Chinese ALS patients should be assessed for confirmation. Overall, it is clear that the mutation spectrum of ALS varies among different ethnic populations^8^.

The p.L39V (L38V) mutation has been reported in some FALS patients and is associated with earlier disease onset (mean onset age: 41.5–44.9 years), a mean duration of 2.8 years and classical ALS symptoms^4^,^35^,^36^,^37^, consistent with the presentation of our patient carrying the p.L39V (L38V) mutation. The p.G42D (G41D) mutation has often been reported in FALS patients and is associated with the initial symptoms of lower limb weakness (mean onset age: 46.0 years), very slowly ascending paresis, and survival for as long as a decade or more^37^,^38^,^39^; this presentation is different from the late onset and shorter survival of our patient carrying the p.G42D (G41D) mutation. This difference might have occurred because the early age of onset predicts longer survival, consistent with the previous observations^37^. The p.H44R (H43R) mutation was first identified in a 58-year-old Japanese female FALS patient who had initial symptoms of lower limb weakness and who died of respiratory failure 7 months after the onset^40^, results similar to the observations in our patient carrying the p.H44R (H43R) mutation except for the negative family history. The p.P67S (P66S) mutation has been reported in a Serbian patient who presented with early onset (21 years) and rapid disease progression (died within 17 months)^34^. Our patient with the p.P67S (P66S) mutation also presented with rapid progression. However, in a South Korean family, patients with p.P67S (P66S) in the *SOD1* gene have been reported to show slower progression^41^. Previous reports have found that FALS patients with p.C112Y (C111Y) and p.I114T (I113T) mutations of the *SOD1* gene show extreme variability in the age of onset, clinical manifestations and disease progression^33^,^37^,^42^,^43^. The p.I114T (I113T) mutation often results in a late age of onset^37^,^44^. A male SALS patient with the p.I114T (I113T) mutation was reported to have the initial symptom of bilateral arm weakness at the age of 64, and this patient died of respiratory failure within 7 months^45^; however, our patient with the p.I114 mutation presented with slow progression with the initial site of the left lower limb.

Aside from these six previously reported mutations, the pathogenic roles of the novel mutations p.G73D and p.V120F were suggested by the following observations. First, the novel variants were absent in our HCs, as assessed using the PCR-RFLP method as well as available repositories including the dbSNP (build 146), the 1000 Genomes database and the Exome Aggregation Consortium (ExAC) database. Second, the amino acids “G” of p.G73 and “V” of p.V120 are highly conserved across species (Fig. 1). We did not find any mutations of other ALS causative genes in patients carrying these heterozygous mutations. Third, two prediction tools, SIFT and PolyPhen-2, predicted detrimental roles of these mutations. Finally, for p.G73D, in the same region, the pathogenic mutation p.G73S (G72S) has been reported in an SALS patient with early onset (28 years) and rapid disease progression (died in 15 months)^46^. As previously noted, G73 is located in the SOD1 zinc-binding sub-loop, which plays a critical role in stabilizing the conformation of SOD1. Changes in amino acids in this region may lead to alterations in zinc-binding activity. Some studies have proposed that abnormalities in this region of SOD1 may underlie its ALS-related toxicity^34^,^47^,^48^. For p.V120F, the onset was very early, suggesting that this mutation may play an important role in the development of ALS. Nonetheless, further studies are required to clarify the pathogenic effects of these mutations.

Currently, the best-studied mechanism for ALS involves SOD1^49^, and more than 170 missense mutations have been reported in *SOD1* (http://alsod.iop.kcl.ac.uk) to date. *SOD1* mutations can give rise to almost all described clinical ALS phenotypes, such as progressive muscular atrophy and bulbar palsy, but no clear correlations between the mutated codon and the phenotype have been found. Disease duration may vary among patients harboring the same *SOD1* mutations^50^, although most of our patients who carried mutations of the *SOD1* gene presented with clinical manifestations consistent with those of previously reported patients^37^. Notably, our patient who carried the p.G42D (G41D) mutation showed a relatively rapid disease progression (16 months) and deviated from the usual very slow progression. The disease phenotype and progression may be influenced by epigenetic factors such as sex, modifier genes, environmental factors, and other unknown factors^51^. Our study found two novel mutations in *SOD1* from two SALS patients who presented with relatively slow disease progression. This observation supported the pathogenic roles of these mutations by primary analysis, though these results were not verified. Therefore, our findings expand the mutation spectrum of the *SOD1* gene in ALS.

In conclusion, mutation of the *SOD1* gene appears to be a common cause of ALS in Chinese individuals. Our findings expand the mutation spectrum of the *SOD1* gene in ALS. Our findings also support the assumptions that the regions of *SOD1* mutations are diverse among different geographical backgrounds and that the mutation spectrum of ALS varies among different ethnic populations.

## Additional Information

**How to cite this article:** Wei, Q. *et al*. Analysis of *SOD1* mutations in a Chinese population with amyotrophic lateral sclerosis: a case-control study and literature review. *Sci. Rep.* **7**, 44606; doi: 10.1038/srep44606 (2017).

**Publisher's note:** Springer Nature remains neutral with regard to jurisdictional claims in published maps and institutional affiliations.

## Supplementary Material

Supplementary Tables

## Figures and Tables

**Table 1:** Demographic and clinical characteristics of cases and controls.

Variable	FALS	SALS	HC
Cases, N	12	487	466
Sex, F (%)	2 (16.67)	200 (41.07)	217 (46.57)
Age (range, years)	43.00 ± 15.18 (18–68)	54.23 ± 11.80 (23–86)	52.78 ± 12.44 (17–82)
Mean onset age (years)	36.56 ± 11.65	52.72 ± 5.39	—
Site of onset (bulbar, %)	2 (16.67)	97 (19.92)	—

**Table 2:** Clinical features of patients carrying sequence variants of the SOD1 gene.

	Sex	Region	Nucleotide change	Variant	Age at onset (y)	Age at examination (y)	Survival time (months)	Initial symptoms	Family history
Case 1	Female	Exon2	c.115C > G	P.L39V	39.7	41.2	24	Bulbar	Yes
Case 2	Male	Exon2	c.125G > A	p.G42D	59.0	59.4	16	Upper limb	Yes
Case 3	Male	Exon2	c.131 A > G	p.H44R	47.5	47.9	9	Upper limb	No
Case 4	Female	Exon3	c.199C > T	p.P67S	47.3	47.8	14	Lower limb	No
Case 5	Male	Exon3	c.218G > A	p.G73D*	44.1	48.8	63	Lower limb	No
Case 6	Male	Exon4	c.335G > A	p.C112Y	38.8	39.7	39 (alive)	Lower limb	Yes
Case 7	Female	Exon4	c.341T > C	p.I114T	54.4	56.1	38 (alive)	Lower limb	No
Case 8	Male	Exon5	c.358G > T	p.V120F*	33.1	34.1	33 (alive)	Upper limb	No

^*^Not previously reported.
