# Supplementary material for: Analysis of SOD1 mutations in a Chinese population with amyotrophic lateral sclerosis: a case-control study and literature review
Source: Sci Rep. 2017 Mar 14;7:44606. doi: 10.1038/srep44606 (PMC5349524; doi:10.1038/srep44606)
Supplement: Supplementary Tables [file srep44606-s1.pdf]

Analysis of *SOD1* mutations in a Chinese population with amyotrophic lateral sclerosis: a case-control study and literature review

QianQian Wei[1], QingQing Zhou[1], YongPing Chen[1], RuWei Ou[1], Bei Cao[1], YaQian Xu[1], Jing Yang[1], Hui-Fang Shang[1]*

[1]Department of Neurology, West China Hospital, Sichuan University, Chengdu, Sichuan, China.

*Correspondence to: Hui-Fang Shang, Department of Neurology, West China Hospital, Sichuan University, 610041, Chengdu, Sichuan, China. E-mail: hfshang2002@163.com, FAX: 0086-028-85423550.

supplementary Table 1: The primer sequences, the length of the PCR products and the restriction enzymes used to be analyzed mutations of the SOD1 gene

Mutation	Exon	Protein	Primers	PCR products(bp)	Restriction enzyme	Fragments (bp)
c.218G>A	3	p.G73D	F: 5' GCACTTTCTCCATGGGAAG 3'<br>R: 5' AGTATACCATATGAACTCCAG 3'	231	HpyCH4III	147+84

supplementary Table 2: Repeat-primed PCR protocol of *C9orf72*.

Repeat-primed PCR Primers			
MRX-F	FAM- 5'-TGTAAAACGACGGCCAGTCAAGGAGGGAAACAACCGCAGCC-3'		
MRX-M13F	5'-GCACGACGTTGTAGGACGACC-3'		
MRX-R1	5'-GCACGACGTTGTAGGACGACCCCGGCCCCGGCCCCGG-3'		
Repeat-primed PCR solution			
10ul Rxn	Item	conc.	Final
1.5	DNA	10ng/ul	15ng
5	2×GC-melt buffer	2×	1×
2	NTP-deaza(1mM A,C,T+1mM deaza dGTP)	5×	0.2mM
0.1	Primer MRX-F	10uM	0.1uM
0.1	Primer MRX-M13F	10uM	0.1uM
0.1	Primer MRX-R1	10uM	0.1uM
0.1	LA taq ploymerase	5U/ul	0.6U
1.1	H_2O		
Repeat-primed PCR Cycling program			
95℃ 10';95℃ 30', 72℃ 5'(35cycles); 72℃ 7'; 4℃ forever			

supplementary Table 3: PCR primers and conditions of *TARDBP*, *FUS*, *PFN1* and *SQSTM1*.

Gene	Exon	Forward primer 5’→3＇	Reverse primer 5＇→3＇	★Annealing temperature(℃)
TARDBP	1	TCAATCTTCAGCTTTTCAGGC	TGCCAGGACCTAACGACGCT	56
	2	GAACTCTGACATGGTTTGGGT	TTTCAGGAGACATTCTGCCA	53
	3	TGCCAAGTTTTCAGTGTCTTA	AGGGAACATAGTGATACCCCA	53
	4	TTAAGCCACTGCATCCAGTTG	CCCTGCCGCTATCTTTTCTAA	53
	5	GGCGAATGATTTTGTTATATC	CGGGACATATCGTTAAGGAGA	53
	6	TGCTTATTTTTCCTCTGGCT	CTCCACACTGAACAAACCAA	59
FUS	1	GTGTTGGAACTTCGTTGCTT	GTCCCACTGAAAACGAAAAG	58
	2	CAGAGTGGCAGCTGAAGATA	GGTAGGCCCCATAGCTAAAT	60
	3	CCCAAAGGTGAGTGCTATTT	GACCAGACTCCGTCTCCA	60

4	CTCTTTCCTGGTGGCTTTT	TCATCAATTCACCCCTCTTT	58
5	TGTTGGGTACAGAGAATGGA	GCAACAGAGACAGAGCAAGA	62
6	GGCACTTGTCAAACCTTTTC	CACTCCCCACCAAAGATACT	60
7	GTTGGAAGCTTCATGTCCTT	GTTCTGGACACCTCAAAACC	60
8	TAACGGCTCATCTTTTCCTT	ATCCAGGCAGTCTTGATACC	60
9	TGATACCAGTTGCTTGATGG	TAAGCTGGCAACAACCACTA	60
10	GAAGAGGGGAGCTGAAGTTT	CCCTCAAACTATGGCTTGTT	60
11	GGCACGCTTCTCTTGTATTT	GCCTTTACCATCTTCTGCAC	60
12	GCATGGAATGGGTTAGATTT	CGCATGCAAGAGAACTAAGA	60
13	AAGGTAATGGATTGGGTTCA	CATATTCCCATTCCCCTATG	58
14	CATGGGTAAGAAAGGCAGAC	CCTATGGCCTCTGTTCAACT	60

	15	TACTCGCTGGGTTAGGTAGG	TGATCAGGAATTGGAAGGTT	60
PFN1	1	GGGAGGAGCAGGAAGTGGCG	CCAGGGCAAGCACCCAGTCA	69
	2	CCCACAGTCCTCAGAGTTTC	AGCACCCTCAAGATTACCAG	63
	3	AGATGAGGTTGGGTTACAGC	GGAGGGATATGGGTAGGG	62
SQSTM1	1	ACCTGGAGCGAGGGGTAGCG	CCATGGCCGAACTGGGGAC	64.5
	2	GCCCTGTGAGTGTCCCTTTC	GGACTAGAAGCATGTACCACCAC	62
	3、4	GCTCTGCTGCCCTCACCTA	CCACTTTGCAGGGTTCTTGT	62
	5	CAGAGTGGGAGGAAGGAGA	TGTCAAGGGCATGTCAATT	62
	6、7	GCCACCATCCAGACACTTAG	TGTCGCTGAAATCAGAGGAG	58
	8	CGCAGTGGCAGAGTTGAGCAG	CAGGGCACCCAGGAAACATCA	67.2

PCR conditions: 95℃ 5’; 95℃ 30’’, annealing★ 30’’, 72℃ 30’’(35cycles);72℃ 5’; 4℃ forever.

supplementary Table 4: The mutation frequencies of common causative genes in Chinese ALS patients.

Patients	Genes	References	Cohort	Total	Mutation number	Frequencies	Total Frequencies
SALS							
	SOD1	Li et.al [2012][1]	Mainland	142	3	2.11%	
		Soong et.al [2014][2]	Taiwan	131	4	3.05%	
		Zou et.al [2015][3]	Mainland	324	3	0.93%	
		Hou et.al[2016][4]	Mainland	158	3	1.89%	
		The current study	Mainland	487	5	1.03%	1.45%[18/1242]
	TARDBP	Xiong et.al [2010][5]	Mainland	71	0	0%	
		Huang et.al [2012][6]	Mainland	165	1	0.61%	
		Soong et.al [2014][2]	Taiwan	131	0	0%	
		Zou et.al [2015][3]	Mainland	324	3	0.93%	
		Ju et.al [2016][7]	Mainland	200	1	0.5%	
		Hou et.al [2016][4]	Mainland	158	0	0%	0.48%[5/1049]
	FUS	Soong et.al [2014][2]	Taiwan	131	2	1.55%	
		Zou et.al [2015][3]	Mainland	324	6	1.85%	
		Hou et.al [2016][4]	Mainland	158	0	0%	1.31%[8/613]

	VCP	Zou et.al [2015][3]	Mainland	324	0	0%	0%[0/324]
	ANG	Zou et.al [2015][3]	Mainland	324	1	0.31%	0.31%[1/324]
	SQSTM1	Chen et.al [2014][8]	Mainland	306	3	0.98%	
		Yang et.al [2015][9]	Mainland	436	6	1.38%	1.21%[9/742]
	PFN1	Chen et.al [2013][10]	Mainland	540	1	0.19%	
		Zou et.al [2015][3]	Mainland	324	0	0%	0.12%[1/862]
	TBK1	Tsai et. al [2016][11]	Taiwan	175	1	0.57%	0.57%[1/175]
	CHCHD10	Zhou et.al [2016][12]	Mainland	487	2	0.41%	
		Jiao et.al [2016][13]	Mainland	150	0	0%	0.31%[2/637]
	C9orf72	Zou et.al [2015][3]	Mainland	324	0	0%	
		Soong et.al [2014][2]	Taiwan	131	2	1.53%	
		Jiao et.al [2014][14]	Mainland	100	0	0%	
		He et.al [2015][15]	Mainland	1062	3	0.28%	
		Chen et.al [2016][16]	Mainland	918	8	0.87%	0.53%[13/2435]
	Total						6.29%
FALS							
	SOD1	Liu et.al [2014][17]	Mainland	8	3	37.5%	

	Tsai et. al [2011][18]	Taiwan	15	3	20%	
	Li et.al [2012][1]	Mainland	7	3	42.9%	
	Zhang et.al [2012][19]	Mainland	43	10	23.3%	
	Soong et.al [2014][2]	Taiwan	30	8	26.67%	
	Zou et.al [2015][3]	Mainland	20	5	25%	
	Hou et.al [2016][4]	Mainland	15	3	20%	
	The current study	Mainland	12	3	25%	25.33%[38/150]
TARDBP	Xiong et.al [2010][4]	Mainland	5	1	20%	
	Tsai et. al [2011][18]	Taiwan	15	3	20%	
	Soong et.al [2014][2]	Taiwan	30	7	23.33%	
	Zou et.al [2015][3]	Mainland	20	0	0%	
	Ju et.al [2016][7]	Mainland	18	1	5.55%	
	Hou et.al [2016][4]	Mainland	15	0	0%	12.24%[12/98]
FUS	Tsai et. al [2011][18]	Taiwan	15	2	13.33%	
	Soong et.al [2014][2]	Taiwan	30	2	6.67%	
	Zou et.al [2015][3]	Mainland	20	2	10%	
	Hou et.al [2016][4]	Mainland	15	2	13.33%	10.00%[8/80]

VCP	Zou et.al [2015][3]	Mainland	20	0	0%	0%[0/20]
ANG	Liu et.al [2014][17]	Mainland	8	0	0%	
	Zou et.al [2015][3]	Mainland	20	0	0%	0%[0/10]
SQSTM1	Yang et.al [2015][9]	Mainland	35	0	0%	0%[0/35]
PFN1	Zou et.al [2015][3]	Mainland	20	0	0%	0%[0/30]
	Chen et.al [2015][10]	Mainland	10	0	0%	
TBK1	Tsai et.al [2016][11]	Taiwan	32	0	0%	0%[0/32]
CHCHD10	Zhou et.al [2016][12]	Mainland	12	0	0%	
	Jiao et.al [2016][12]	Mainland	15	0	0%	0%[0/27]
C9orf72	Soong et.al [2014][2]	Taiwan	30	5	16.67%	
	Liu et.al [2014][20]	Mainland	62	1	1.61%	
	Jiao et. al [2014][14]	Mainland	5	1	20%	
	Zou et.al [2015][2]	Mainland	20	0	0%	5.98%[7/117]
Total						53.55%

Supplementary references

1. Li XG, Zou ZY, Peng Y, Lin YC, Zhang JH, Xie MQ, Zhang LH, Liu MS, Cui LY. Molecular genetics of amyotrophic lateral sclerosis (2012). Medical Journal of Peking Union Medical College Hospital 2012;3;337-43.
2. Soong BW, Lin KP, Guo YC, Lin CC, Tsai PC, Liao YC, Lu YC, Wang SJ, Tsai CP, Lee YC (2014) Extensive molecular genetic survey of Taiwanese patients with amyotrophic lateral sclerosis. Neurobiology of aging 35 (10):2423.e2421-2426. doi:10.1016/j.neurobiolaging.2014.05.008
3. Zou ZY, Liu MS, Li XG, Cui LY (2015) The distinctive genetic architecture of ALS in mainland China. Journal of neurology, neurosurgery, and psychiatry. doi:10.1136/jnnp-2015-311654
4. Hou L, Jiao B, Xiao T, Zhou L, Zhou Z, Du J, Yan X, Wang J, Tang B, Shen L (2016) Screening of SOD1, FUS and TARDBP genes in patients with amyotrophic lateral sclerosis in central-southern China. Sci Rep 6:32478.
5. Xiong HL, Wang JY, Sun YM, Wu JJ, Chen Y, Qiao K, Zheng QJ, Zhao GX, Wu ZY (2010) Association between novel TARDBP mutations and Chinese patients with amyotrophic lateral sclerosis. BMC medical genetics 11:8. doi:10.1186/1471-2350-11-8
6. Huang R, Fang DF, Ma MY, Guo XY, Zhao B, Zeng Y, Zhou D, Yang Y, Shang HF (2012) TARDBP gene mutations among Chinese patients with sporadic amyotrophic lateral sclerosis. Neurobiology of aging 33 (5):1015.e1011-1016. doi:10.1016/j.neurobiolaging.2010.07.007
7. Ju X, Liu W, Li X, Liu N, Zhang N, Liu T, Deng M (2016) Two distinct clinical features and cognitive impairment in amyotrophic lateral sclerosis patients with

TARDBP gene mutations in the Chinese population. Neurobiology of aging 38:216.e211-216. doi:10.1016/j.neurobiolaging.2015.10.032

8. Chen Y, Zheng Z-Z, Chen X, Huang R, Yang Y, Yuan L, Pan L, Hadano S, Shang H-F (2014) SQSTM1 mutations in Han Chinese populations with sporadic amyotrophic lateral sclerosis. Neurobiology of aging 35 (3):726.e727-726.e729. doi:10.1016/j.neurobiolaging.2013.09.008

9. Yang Y, Tang L, Zhang N, Pan L, Hadano S, Fan D (2015) Six SQSTM1 mutations in a Chinese amyotrophic lateral sclerosis cohort. Amyotrophic lateral sclerosis & frontotemporal degeneration 16 (5-6):378-384. doi:10.3109/21678421.2015.1009466

10. Chen Y, Zheng ZZ, Huang R, Chen K, Song W, Zhao B, Chen X, Yang Y, Yuan L, Shang HF (2013) PFN1 mutations are rare in Han Chinese populations with amyotrophic lateral sclerosis. Neurobiology of aging 34 (7):1922.e1921-1925. doi:10.1016/j.neurobiolaging.2013.01.013

11. Tsai PC, Liu YC, Lin KP, Liu YT, Liao YC, Hsiao CT, Soong BW, Yip PK, Lee YC (2016) Mutational analysis of TBK1 in Taiwanese patients with amyotrophic lateral sclerosis. Neurobiology of aging 40:191.e111-196. doi:10.1016/j.neurobiolaging.2015.12.022

12. Zhou Q, Chen Y, Wei Q, Cao B, Wu Y, Zhao B, Ou R, Yang J, Chen X, Hadano S, Shang HF (2016) Mutation Screening of the CHCHD10 Gene in Chinese Patients with Amyotrophic Lateral Sclerosis. Molecular neurobiology. doi:10.1007/s12035-016-9888-0

13. Jiao B, Xiao T, Hou L, Gu X, Zhou Y, Zhou L, Tang B, Xu J, Shen L (2016) High prevalence of CHCHD10 mutation in patients with frontotemporal dementia from China. Brain : a journal of neurology 139 (Pt 4):e21. doi:10.1093/brain/awv367

14. Jiao B, Tang B, Liu X, Yan X, Zhou L, Yang Y, Wang J, Xia K, Shen L (2014) Identification of C9orf72 repeat expansions in patients with amyotrophic lateral sclerosis and frontotemporal dementia in mainland China. Neurobiology of aging 35 (4):936.e919-922. doi:10.1016/j.neurobiolaging.2013.10.001
15. He J, Tang L, Benyamin B, Shah S, Hemani G, Liu R, Ye S, Liu X, Ma Y, Zhang H, Cremin K, Leo P, Wray NR, Visscher PM, Xu H, Brown MA, Bartlett PF, Mangelsdorf M, Fan D (2015) C9orf72 hexanucleotide repeat expansions in Chinese sporadic amyotrophic lateral sclerosis. Neurobiology of aging 36 (9):2660.e2661-2668. doi:10.1016/j.neurobiolaging.2015.06.002
16. Chen Y, Lin Z, Chen X, Cao B, Wei Q, Ou R, Zhao B, Song W, Wu Y, Shang HF (2016) Large C9orf72 repeat expansions are seen in Chinese patients with sporadic amyotrophic lateral sclerosis. Neurobiology of aging 38:217.e215-222. doi:10.1016/j.neurobiolaging.2015.11.016
17. Liu ZJ, Li HF, Tan GH, Tao QQ, Ni W, Cheng XW, Xiong ZQ, Wu ZY (2014) Identify mutation in amyotrophic lateral sclerosis cases using HaloPlex target enrichment system. Neurobiology of aging 35 (12):2881.e2811-2885. doi:10.1016/j.neurobiolaging.2014.07.003
18. Tsai CP, Soong BW, Lin KP, Tu PH, Lin JL, Lee YC (2011) FUS, TARDBP, and SOD1 mutations in a Taiwanese cohort with familial ALS. Neurobiology of aging 32 (3):553.e513-521. doi:10.1016/j.neurobiolaging.2010.04.009
19. Zhang HG, Tang L, Zhang N, Fan DS. Phenotype and genotype of mutations in SOD1 mutation in amyotrophic lateral sclerosis of Chinese origin. J Chin Neurol (Chinese) 2012;45;453-8.
20. Liu R, Tang L, Cai B, Liu X, Ye S, Ma Y, Zhang H, Fan D (2013) C9orf72 repeat expansions are not detected in Chinese patients with familial ALS. Amyotrophic lateral sclerosis & frontotemporal degeneration 14 (7-8):630-631. doi:10.3109/21678421.2013.817588
